# Striving to provide conditional access: strategies parents use to mediate the screentime of their children with autism spectrum disorder

**DOI:** 10.3389/frcha.2025.1540147

**Published:** 2025-08-20

**Authors:** Faatima Ebrahim, Pamela Gretschel, Iesrafeel Abbas

**Affiliations:** Division of Occupational Therapy, Department of Health and Rehabilitation Sciences, Faculty of Health Sciences, University of Cape Town, Cape Town, South Africa

**Keywords:** screen time, autism spectrum disorder, parental strategies, qualitative, restrictive mediation, LMIC

## Abstract

**Introduction:**

There has been a growing presence of screentime, in the lives of children, with an escalation in use during the COVID-19 pandemic restrictions. Children with autism spectrum disorder show a particular preference for engagement in screentime. Gaining parental understandings of the steps they take to mediate excessive screentime can assist in developing interventions which mitigate the well documented negative impacts of screentime for children with autism spectrum disorder. This paper presents the findings of a study which explored parental perceptions of the screentime use and the strategies parents used to manage the screentime engagement of their children with autism spectrum disorder.

**Methods:**

A qualitative descriptive design, using semi-structured, in-depth interviews with seven purposively selected parents, was used to achieve the above objectives. Data was thematically analysed using an inductive approach.

**Results:**

One of the four themes generated during the study; Striving to provide conditional access to screentime details the varied mediation strategies parents used to manage their child's screentime under the two categories of Content monitoring and Setting limits.

**Discussion:**

The findings of this study, describing the various restrictive strategies parents use to manage the screentime use of their children, were comparable to prior studies. Findings that built on existing evidence, describe the strategies parents used i.e., distraction and preparing for the cessation of screentime, to manage screentime in a way that avoided negative behaviour in their child and parental stress linked to this behaviour. It is certain, that screentime will remain a predominant occupation for children with autism spectrum disorder therefore, early childhood interventionists need to consider how to optimize the nature of engagement of screentime.

## Introduction

The occupation of screentime (ST) in children has gained worldwide attention as its prevalence increases in the lives of children. Studies demonstrate how neurotypical children are far exceeding the recommended user guidelines of 30 min to a maximum of 2 hours per day on devices (1, 2). This issue is particularly pronounced in children with autism spectrum disorder (ASD) in North America, Europe and Asia, who on average spend up to 3 hours more on ST per day when compared to neurotypical children (3). This increased vulnerability to excessive ST may be attributed to the characteristics of ASD which includes symptoms such as persistent deficits of social skills and communication, interaction, impulse control, ritualistic, repetitive patterns of behaviour and actions, as well as restricted interests (4, 5). Furthermore, children with ASD are being exposed to devices with screens at increasingly earlier ages (6). The rapid and sometimes repetitive audio-visual stimulus that ST provides, as well as how it reduces the need for social engagement and communication with others, makes ST an interesting and appealing occupation for children with ASD (7, 8).

Excessive ST has been associated with speech and language delays, sleep disturbances, sedentary behaviour, reduced physical activity and other health problems in children with ASD (4, 9). Additionally, the high use of ST may result in an imbalance in time spent on other occupations needed for childhood play, learning and development. Since children with ASD typically have difficulty with impulse control, self-regulation and tend to become fixated on their interests resulting in increasing risk for excessive ST, they require parental involvement to help them moderate their environments (10).

Existing evidence suggests that parents may be exacerbating ST. Parents allow their children with ASD to spend increasing amounts of time on devices with screens, media, and technology (4, 11, 12). In addition, parents' experiences of the positive impact of ST and their beliefs that it aids in the development of their children with ASD could be contributing to its excessive use (1, 13). In contrast, other studies have found that even when parenting views include negative beliefs about ST, the parents still had trouble setting boundaries for its use (14). Parents expressed helplessness when trying to regulate its use, due to the adverse behavioural issues elicited in their child when they tried to control ST (15, 16). This emphasizes the need to understand parental perspectives of ST, the strategies they use, as well as the impact of these strategies to mediate the ST use of their children with ASD.

Parental insight of ST was identified as being essential towards creating effective management strategies for children with ASD (17). After being educated on the risks of excessive ST, 26 parents of children with ASD were able to apply newly learnt ST guidelines and create a family media use plan which not only led to a significant reduction in children's ST but also subsequent improvement in their social functioning, enhancing their social communication. Another study investigating parental knowledge, attitudes and concerns toward ST of children with ASD (*n* = 92) in India, revealed that parents possessed the insight into the potential negative effects of ST on their children (16). These parents restricted their children on devices by setting time limitations, engaging them in other occupations such as outdoor play, selecting the content their children accessed and only allowing access under parental supervision or during specific periods/tasks during the day such as mealtimes (16). These strategies were found to be useful in managing ST of ASD children in contexts outside of South Africa.

In middle-income countries like South Africa, specialized ASD intervention services are extremely limited due to human resource constraints and overburdened and financially constrained public systems (18). Parental involvement thus becomes an important focus of interventions and can be seen as an effective way of providing support for children with ASD (19). Furthermore, parental mediation of ST was identified as a key intervention strategy for children with ASD, in a meta-analysis study on media guidance (17). This puts emphasis on the role and need for parents to provide restriction, active mediation and co-viewing to manage its effects. Exploring parental strategies to mediate ST is an enactment of family centred practice and an intervention approach which can help develop sustainable methods to manage potentially negative impacts of ST (20). Gaining information on the specific details of how they mediate ST has the potential to inform intervention guidelines for better management of ST and for optimizing its use.

This research study then aimed at identifying parents' perceptions of ST of their young children diagnosed with ASD in order to answer the question: What are parents' perceptions of ST of their young children diagnosed with autism spectrum disorder? The objectives included identifying parents' understandings of how and why their children use screen-based media, as well as the effect of ST on their children's lives. A further objective was added to identify and describe the strategies they used to manage ST use. In this paper we present only one of four themes emanating from the data i.e., The theme Striving to provide conditional access to screentime which describes the strategies parents used to manage the ST of their children with ASD.

## Methods

### Methodological approach

A qualitative descriptive research (QDR) design was used to explore and generate an interpretative description of parental perceptions of the engagement of their children with ASD in screentime, inclusive of the strategies they used to mediate ST use (21). QDR allowed the researcher to describe and interpret data that emerged in a way that stayed closer to its description of the participants' perspective (21). QDR was appropriate in this study as it also contributed to emerging research as little is known about the construct of ST within SA.

### Participants

The study was conducted in Cape Town, in the Western Cape Province of South Africa (SA), where although specialized education and services for ASD exist, they are often very limited in accessibility (22, 23).

Parents of children with ASD were recruited via purposive sampling with the assistance of a non-profit organization (NPO) providing various support services, counselling and advocacy for persons affected by ASD. The researcher briefed the NPO on the specific study requirements and participant selection criteria, and the NPO then contacted potential participants meeting the inclusion criteria to determine their willingness to be approached by the researcher to take part in the study. Inclusion criteria were as follows: English or Afrikaans speaking mothers and/or fathers of children aged eight years or younger with a formal diagnosis of ASD, living with the child who had access to ST devices. Participants were excluded if they were actively participating in any current research studies related to their children with ASD. Other caregivers such as grandparents or relatives of the child were excluded from the study due to the focus being primarily on the role of parents.

The profiles of ten potential participants meeting the above selection criteria were shared with the researcher, who then applied the principle of maximum variation (24) to select a sample of socio- economic and culturally diverse parents living in different geographical settings in Cape Town. See Table 1 for demographic information. A broad range of ages of children were also included in order to provide maximum variation in perspectives. The researcher contacted these participants telephonically to explain the purpose of the study and to obtain their verbal informed consent. Informed consent forms were signed by participants and returned, either via email or in person before data collection commenced. A mutually convenient meeting time and online/in-person venue of the participants choice was organized for the interviews. All names are pseudonyms.

**Table 1:** Participant demographic details outlining location and children.

Participant	Anna	Fran	Beth	Molly	Tumi	Cynthia	Ezra
Geographical Location	Blouberg	Grassy Park	Eesterivier	Mitchell’s Plain	Milnerton	Durbanville	Mitchell's Plain
Ethnicity	Indian	Coloured	Zulu	Coloured	Xhosa	White Afrikaans	Zimbabwean foreign national
Participant in this study	Mother	Mother	Mother	Mother	Mother	Mother	Father
Occupation of participant	Part time teacher	Stay at home mother	Nurse	Student	Stay at home Mother	Part time works from home	Full time employed sales
Age of child with ASD (years and months)	5 years	4 years 6 months	7 years 1 month	7 years 11 months	6 years	6 years 7 months	2 years 10 months
Sex of child	Male	Male	Male	Female	Male	Male	Female
Age of other children	8 years	11 years & 7 years	16 years	4 years	8 years	17 years	None
Resides with	Husband & children	Husband, children & mother-in- law	Boyfriend & children	Husband & children	Child only	Husband & children	Wife & child
Primary caregiver	Both parents	Both parents but mostly mother	Both parents, older sibling & neighbour	Both parents	Mother & mothers’ sister	Both parents, two domestic workers, maternal & paternal grand parents	Both parents

### Procedure

Individual face to face, and online in depth semi structured interviews of 90 min each were conducted with the participants. The interviews were completed and transcribed over a period of two months. The interview guide was informed by previous literature on ST and included introductory questions related to the participants' context and background, as well as study specific questions that focused on eliciting information related to the research aims and objectives (see Appendix A). A parent of a four- year-old child with ASD attending a local special needs school, reviewed the interview guide and suggested to the inclusion of the additional questions to elicit more information about what was being asked. This feedback was used to refine the interview guide. The question “How does your child engage and interact with others in your family?” was added after it was found that the original question “How does your child communicate with you?” required more probing. All interviews were conducted in English. All face-to-face interviews were audio recorded on the researcher's password protected smartphone and a backup audio recorder on her password protected laptop. All online interviews were recorded using password protection on the Zoom app and were uploaded immediately post-interview, to the researcher's laptop as well a password protected storage on an online collaboration and learning system. Data was deleted from the researcher's smartphone. The researcher kept a personal notebook/journal to record field notes that could be used as data, that included points of significance brought up during the interviewing process, as well as her reflections on these points. The researcher contacted participants telephonically and via email to do member checking. All participants confirmed the researcher's interpretations, and no changes were made.

### Data analysis

**Figure 1:**
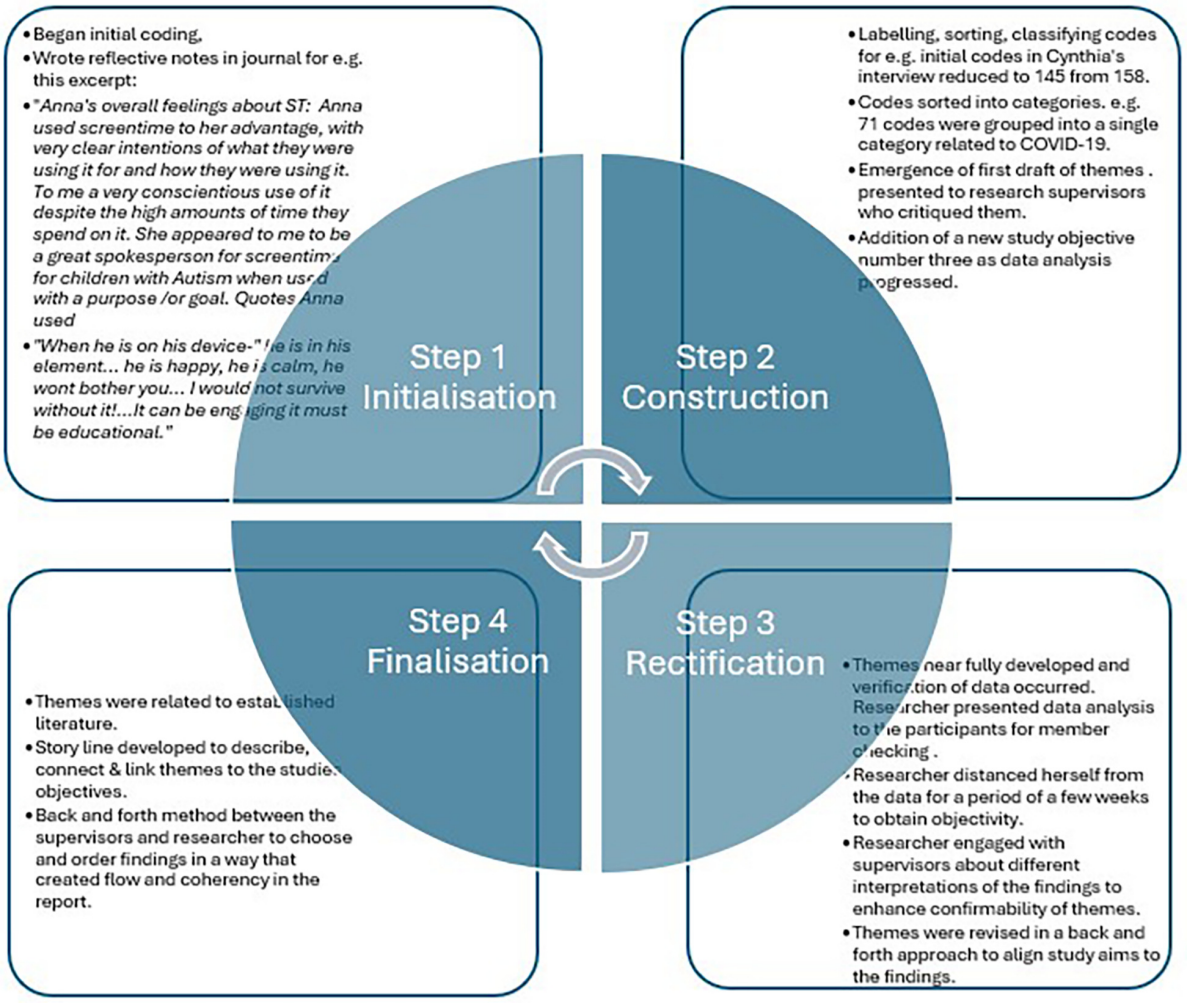
Phases of thematic analysis that were applied in this study with examples.

Audio data was transcribed verbatim using software on the researcher's laptop called Descript^©^ (25) within seven days after each interview. Accuracy of transcripts were verified by the researcher who listened to the audio recordings whilst reading transcriptions repeatedly. Thematic analysis using an inductive approach was applied through four phases of initialization, construction, rectification and finalisation (26). A qualitative data analysis software program, nVivo12 was used to organize the coding. During the initialization phase transcriptions were manually coded line by line by the researcher highlighting units of meaning. The researcher looked for abstractions in participants' accounts and wrote reflective notes through journalling whilst examining her own assumptions and bracketing her ideas. Following from this, the construction phase involved an iterative process between the researcher and co- authors who discussed the data findings and where data was then assimilated, compared and labelled into categories to identify potential main themes. During this stage it was established that data saturation had occurred after seven interviews as no new ideas and codes were emerging. The process of rectification began where emerging themes were reviewed in consultation with both research supervisors examining different interpretations to enhance confirmability. The researcher then contacted participants to review the data and to do member checking. Participants agreed that the interpretations were representative of their views. This phase also included immersion into the data followed by a period of distancing the researcher from it to ensure objectivity. Finalisation was then completed through constant consultation with research supervisors (see Figure 1).

### Trustworthiness

All aspects of trustworthiness were fully considered in this study, through the use of peer debriefing with the researcher's supervisors, member-checking, use of direct participant quotes, a detailed audit trail, reflective journaling, purposive sampling and detailed descriptions of the participants, their contexts, and their multiple viewpoints.

### Research ethics

Ethical clearance was granted from the University of Cape Town (UCT) Faculty of Health Sciences Human Research Ethics Committee (HREC 253/2021) and all ethical considerations of informed consent, confidentiality, anonymity and beneficence was adhered to (27). The researcher identified that some parents required further information, resources and advice for their child with ASD. In these cases, the researcher recommended support services that parents could access. The researcher reassured participants that the study did not aim to evaluate or judge their decisions regarding ST. When recalling and describing events around ST of their children, some parents relived negative emotions brought up by these memories, leading to emotional distress. The researcher provided containment through displaying empathy in these instances and also made appropriate referrals to AWC, Lifeline Western Cape and Western Cape Health Department for further support when necessary.

## Results

See Table 2 for an overview of all the themes that emerged from this study. Not all themes in Table 2 are reported in this paper. [For a full report on all themes please refer to the complete thesis available on open source online (28)].

**Table 2:** All themes and categories from this study. Theme 3 presented in this article is highlighted.

Theme	Category
Theme 1: Seeking out ST opportunities	• Child's ST preferences and patterns of use • Limited interest in non-ST activities
Theme 2: ST, a mixed blessing	• Benefits of ST • Drawbacks of ST
Theme 3: Striving to provide conditional access to ST	• Content monitoring • Setting Limits
Theme 4: COVID-19 “intensified the whole screen time business”	• ST as an appealing replacement • New challenges in managing ST

Theme one, *seeking out ST opportunities*, highlighted the time use, functionality and content of ST. This described parents' reports as patterns and preferences of how their children use a wide variety of time, devices, and content in line with their interests, as well as how their limited interest in non-ST activities cause them to seek out opportunities for ST. Theme two, *ST, a mixed blessing*, confirmed the literature on how parents perceive benefits of ST being a tool for learning, self-regulation, parent child interaction and respite and perceive drawbacks of ST to increase challenging behaviour, dependency, negative feelings and interfere with social engagement. Parents expressed grappling with ST, as it created many opposing effects on their children's lives often leaving them conflicted. The fourth theme, *COVID-19 “intensified the whole screen time business”* identified and described how the COVID-19 pandemic marked the onset of many changes to ST for children with ASD. These ranged from increased time on devices, to accessing of new and voluminous content through the introduction of the internet and digital online schooling. The changes resulted in ST becoming an appealing replacement whilst presenting new challenges that parents experienced managing its use during the lockdown restrictions. The Theme three (presented in detail in this section*), Striving to provide conditional access to ST*, described how parents endeavoured, via the strategies of content monitoring and setting limits, to curb their child's excessive use of ST. Their use of these strategies was driven by their perceptions of the potentially negative impact of ST on their child's behaviour.

### Theme three: striving to provide conditional access to ST

#### Content monitoring

Parents took on the role of consciously and deliberately monitoring the content that their child accessed.

If you monitor it correctly and you, you block the things that you don't want, your kids and you all, both the phones are linked directly to my husband's email account. (Anna, childs age: 5yrs)

Ezra was very specific about the type of content that his child was allowed to access, limiting it to only ST that served educational purposes.

We decide what she watches because what we do is we want her to learn something. (Ezra, childs age: 2yrs 10 mnths)

It's only educational stuff. So, like my husband and I won't put on, a TV show for adults….but I do put, the science shows, planets, you know, like the planet discovery, planet channel. Yes. Educational stuff. ….but it is entertaining and educational at the same time. (Anna, child's age: 5yrs)

Parents downloaded a list of programs that they had curated to be acceptable for the child to engage with, and the child was given the freedom to choose from this list eliminating the need for continuous monitoring.

I don't use streaming online Netflix and YouTube. And then my husband downloads like, sign language, videos, and stuff for him. And so, when I say that, like we leave it on, we leave the TV on. There's always something that is able to educate him on this, even though it's always on. (Anna, childs age: 5yrs)

Cynthia noted that although she was vigilant about her child's ST content it was a growing challenge to monitor the considerable quantity of it from streaming platforms like YouTube which keeps adding new content via its algorithms.

I need to check that he doesn't watch things that he's not supposed to, but I mean, that is a bit more difficult now with a normal YouTube than the kids YouTube. (Cynthia, child's age:6yrs, 7mnths)

#### Setting limits

Setting limits was another strategy used by the parents in a direct or indirect manner to manage ST. Indirectly parents set limits by offering gentle encouragement and the promise of other appealing activities to coax the child away from the device with minimal fuss.

He does sometimes go into his own little worlds, you know, when he's there, but then yeah. I say: come let's switch off your tablet, please. quickly go and we'll go outside, or we'll go do something. You know, (Dad) says,” come and help me, come, and help me make a fire quickly. We're going to braai” or whatever. And then he's like, okay, he leaves it and let's go. (Cynthia, child's age: 6yrs, 7mnths)

Many parents stated that they only allowed access to ST provided that other activities were also done by the child.

No, I'm not just putting it in his hands and leaving it, you know, he must still do what he needs to do. We, you know, and, and, you know, interact with us when it's supper time and he'll tell me, oh, you making dinner, you know, when is dinner? (Cynthia, child's age: 6yrs, 7mnths)

I will tell her: You first have to do what you can. like for example, when you come from school I will tell her, okay. You first have to eat, undress you. eat and then, um, then you first have to do your homework work if there's any homework and then only. So, I would see that as a way of managing it (ST), and then I will tell her like, until three, until four to go play (Using ST). (Molly, child's age: 7yrs 11mnths)

Some parents also explained how they set limits directly by switching off the devices for their children thereby restricting access.

For example, that she wants to watch something, we can determine that no, it's not the right time. Right. Or if we see it fit, we can say no! (Ezra, child's age:2yrs 10mnths)

Similarly, five parents used a firmer approach such as implementing a “no phones rule.” Where devices were not allowed to be used at all during certain times, for e.g., Sunday outings, mealtimes, near sleep time.

So, weekends, we go for drives and so on. So, then we make it, a rule, there's no phone. (Fran, child's age: 4yrs,6mnths)

Every night at eight, at eight’ o clock the phones go away. Everything goes away because my oldest son has to go to bed for school the next day. (Anna, child's age: 5yrs)

Parents shared that despite their intention to set limits, this was often a challenge. Beth expressed a lack of confidence in her ability to manage ST elsewhere during the day except when she used the child's daily routines to implement the “no phone rule”.

I don't know what I can do if maybe they, there is someone who can like recommend, come show me how I can make things. a bit different So I would love that!…………When he must go to sleep or supper time, he knows that he must switch it (the phone) off. We are having a meal as a family. So, he must do. (Beth, child's age 7yrs 1mnth)

Using a direct approach such as the “no phone rule” was challenging for Molly. She described how she avoided the conflict associated with this direct approach, by adopting an alternate more indirect approach.

It's the only way I can manage it really, is for the Wi-Fi to be switched off that's, that's the only way other than that, like the first day, like if we tell her, like, the Wi-Fi is off then she'll like “why is the Wi-Fi off?” but when it comes to, comes to that two that three (days without it) then she only settles. ok its off! (Molly, child's age 7yrs 11mnths)

Other ways of managing ST whilst trying to avoid conflict, included telling fibs about devices not being available, limited access to wi-fi or hiding devices from the child in order to limit usage. These included telling the child that the battery was dead or that data was used up.

My middle child will ask “mommy, can I watch on the phone? so, he will have to come downstairs, because the minute he (Child with Autism) sees the phone, he wants the phone. (Fran, child's age: 4yrs,6mnths)

Many parents described how limits were applied by giving time warnings to help the child transition to leave the device or prepare them to switch it off.

Because he will say he'll watch his device and when I see it's, okay, it's close to the time (bedtime) I say, listen, one more… (episode). Then last one then…, then, it's time that we're going to go to bed and whatever. And then, lately it's just, “I'm done now.” (Cynthia, child's age,6yrs 7mnths)

Similarly, Tumi described how preparing her child, providing him with a step-by-step transitional approach to ending ST, led to him being more willing to terminate the use of his device, without undue distress.

You cannot just say, put down the phone. It's bedtime, put down the phone. It's bath time, put down the phone. We are going, No, you do not do that. You have to prepare him. Boy, in 10 ten minutes, putting down the phones, it's going to be bedtime boy in five minutes, putting down the phones and it's going to be bath time. That's how I normally do. And it's worked for me. And now boy, you only have one minute left. (Interview Tumi.child's age 6yrs)

Fran adopted a similar preparatory approach, drawing on the bedtime routine to prepare the child to hand over the device willingly.

After we brush his teeth and change his nappy, then I'll say, we're going to sleep now. Give me the phone now and he'll hand it to me he understands that. (Fran, child's age: 4yrs,6mnths)

## Discussion

Parents in this study described different ways of mediating ST for their children, through content monitoring and setting limits. These two strategies represented their provision of access to ST, only under certain conditions. Parental mediation of ST has been defined by Valkenburg, 1999 (29) and Warren, 2001 (30), as strategies that parents use to control, supervise, or interpret the screen media that their children have access to. They describe three types of mediation: restrictive, social and active.

Restrictive mediation involves setting rules for the amount of time allowed for ST and for the type of content that can be accessed (29). In this study, parents used content monitoring to mediate ST use. They actively chose, selected, and/or curated content that they deemed appropriate for their child to access. Their deliberate selection of content and vigilance on what was being accessed by their children, are examples of restrictive mediation strategies found in other literature (12, 16). Similarly, restrictive mediation was also reported in a study by Kuo et al,2015 (31), to be a frequently used strategy of parents with children diagnosed with ASD, who reported restricting their video gaming activity and television viewing. Although their study investigated older adolescent children with ASD who used video gaming (content which was not found to be accessed by the younger children in the current study), the strategy to monitor the content of what their children were accessing is similar in this study. Furthermore, setting limits was another restrictive mediation strategy used by parents in this study. Parents reported how they used the “no phones rule” during certain activities or times during the day in order to limit its use. Restricting ST access specifically during meal or bedtimes was previously reported in another study as an effective way to reduce time spent on devices (16). In addition, the findings in this study described another way of limiting use that included how parents allowed children access to devices on condition that they participated in other activities like homework or play, either before or after ST access. This way of reinforcing desired behaviour was also found to be used by parents in previous studies (13, 32). Allowing conditional access to ST as a form of compromise could be regarded as a possible intervention strategy for managing participation and behaviour of children with ASD to engage in other activities and to mediate ST.

In this study parents also used technical restrictions (TR) to mediate their child's ST, for example by accessing YouTube kids which already has built in safety parameters for children or through linking their children's devices to their own in order to set safety controls. TR is defined as using parental controls that are built into devices and software settings that allow parents to monitor, regulate or block content and time (33). This was reported to be used in a previous study that found that parents of neurotypical children under the age of eight years frequently used restrictive mediation that included TR (16). However, none of the parents in the current study reported that they used the time limit controls that automatically turn devices off after a predetermined period of use. Possible reasons for this could be linked to a previous study by Polfuss et al, 2016 (14) conducted in the USA, who also reported this finding and discovered that parents of children between the ages of six-16 years with ASD did not put time limits on their children's ST use when it was used as a way to avoid negative behaviours such as meltdowns. Avoiding negative behaviours from their child was highlighted in a review of literature about risks and protective factors for stress self-management in parents of children with ASD (34). As children with ASD frequently respond aberrantly to changing environments, parents develop approaches to manage their children and their own stress levels caused by these responses to alleviate some of this caregiving burden. Moreover, Polfuss et al,2016 (14) reported that when parents perceived ST as being beneficial for their child, they were less likely to enforce time limits.

Less restrictive mediation strategies were found to be used when parents had positive attitudes to ST like perceiving it to be beneficial for the child (31, 33). Since children with ASD have difficulty with sudden transitioning between activities and require time and preparation to adjust (35), the sudden switching-off of devices might prove too challenging for this group of children resulting in unwelcomed meltdown behaviour that parents would want to avoid. Parents in the current study reported how they used additional restrictive mediation strategies that allowed for a more willing transition to other activities. These included verbally giving time warnings like the “five-minute-rule” limit to prepare the child to switch off the device on their own or hand it over to the parent. This strategy was not found in the literature search. Further examples of restrictive mediation strategies not previously reported in any literature that were less disrupting to the child, included how parents used distraction and telling fibs in setting limits to ST. Distraction was reported in this study as a way to gently persuade the child to leave the device and move onto other activities. Similarly, telling the child fibs or lies about the battery being dead or the data having run out allowed the parent to limit its use whilst avoiding conflict and negative behaviours. Negative behaviours related to restrictive mediation were found to be a notable source of parental stress (31, 36). This was also found in this study and reported on under a different theme: ST, a mixed blessing, that described the perceived benefits and drawbacks of ST and could suggest practical ways for parents to reduce the stress they may experience on a daily basis as a result of wanting to manage ST.

Another mediation strategy reported in the literature as social mediation (SM) included co-viewing or co-playing for entertainment purposes, was also found to be used in this study. In a previous study SM was also applied by parents of young children with ASD (33). However, in the current study co-viewing was not only identified as a ST management strategy but was also described as a benefit of ST that aided in the interaction between the child and the family.

In addition, the use of active mediation (AM) that includes talking to the child about the content accessed was not reported to be used by any of the parents in this study. Kuo et al,2015 (31), found that AM -discussing content and giving explanations or instructions to the child to enhance safety and stimulate learning, was used most frequently through supervising program viewing and giving instructions to adolescent children with ASD. In the current study, this lack of finding could have been attributed to the younger ages of the children i.e., 2 years to 8 years, as well as the lower verbal and language capabilities of each child possibly indicative of their severity of ASD symptoms and developmental stages. However, an examination of the links between the study of age, severity of ASD symptoms and ST use and mediation were, not part of the objectives of this study. Furthermore, the advancement of technology such as augmented reality storybooks, which involve a fusion of storybooks with digital content could possibly create opportunities for parents to implement AM as they co-view and read the stories along with their young child with ASD. In this way active mediation could stimulate learning and manage ST.

## Conclusion and recommendations

Parents attempted to implement a variety of strategies to manage and allow their children ST access, most frequently, only under certain conditions. Furthermore, the findings from this study also builds on existing literature describing how parents try to avoid stress caused through limiting ST by using negative behaviour- avoidance techniques such as distraction and preparation to assist their children to engage in various other activities.

The findings in this study imply that ST is a prevalent and significant occupation in the lives of families and children with ASD. Therefore, assessment by occupational therapists and other early childhood practitioners needs to include an exploration of ST use by these children and their families so that interventions can be directed accordingly if there are concerns. Early childhood practitioners are in a position to provide intervention that supports parents to promote balance between different activities in their children's lives. This should include recommendations to use mediation strategies that emerged from this study and emphasize the importance of parental involvement in the monitoring of ST use of their children. Moreover, clinical practice needs to include advocacy for the accessibility and availability of options for leisure time such as sport, education, and social opportunities for this population. Considering how much technology is part of children with ASD's everyday lives and of such a valued interest to them as reported in this study, it is also recommended that attention be directed into how to incorporate ST as a therapeutic modality. For example, using learning software, as a means towards achieving therapy goals or as a way to facilitate children's participation and engagement in activities and education that they need to do but may not be interested in. In doing so, practice would support the drive towards Sustainable Development Goal Four: Quality Education for the inclusion of persons with disabilities (37), specifically within the early childhood development context.

## Future research, policy and practice

Although learning was reported to be a perceived benefit of ST in this study, none of the parents used any autism specific applications for this purpose. Considering the growing availability of mobile technology especially in South Africa, the potential to use it for wellbeing and learning intervention especially in contexts where there are limited health and educational resources in this population remains largely unexplored and underutilized. Therefore, further investigation into this functionality for educational use as well as for therapeutic use is recommended.

## Limitations and strengths

This study was based on parental perspectives (mostly maternal) of the ST of children with ASD which may have offered limited viewpoints. The study population was restricted to that of the greater Cape Town area and may not have reflected the full spectrum of languages and contexts within South Africa. Furthermore, participants were selected from the pool of clients that were already in contact with the recruiting body and excluded those who did not have access to their services. In keeping with qualitative descriptive research design requirements, the sample was diverse and offered multiple perspectives, however it remains relatively small. In addition, the data collected did not reflect the details of autistic features of the sample which may have offered further insight into the choices parents made in their management of ST.

Parents of children with ASD are often stigmatized in the community and judged for their parenting approaches especially in relation to how much ST their children are allowed (38, 39). This study gave voice to this often-marginalized group describing how they endeavoured to mitigate ST use highlighting their lived experiences and many challenges with parenting their child with ASD. This study sample included a diverse sample of participants representing variation in ethnicity, ages of children, socio-economic status, culture, and belief systems within the CT area of SA. Despite the limitations of this context, the findings of this study confirmed similarities of what was found in other studies globally. Furthermore, these findings builds on existing evidence, describing the strategies parents use i.e., distraction and preparing for the cessation of ST, to manage ST in a way that avoided negative behaviour in their child and parental stress linked to this behaviour.

## Data Availability

The datasets presented in this article are not readily available due to participant confidentiality. Requests to access the datasets should be directed to Faatima Ebrahim, faatima.ebrahim@uct.ac.za.

## Appendix A

Study specific directed questions

*Objective 1: To identify and describe parents understanding of how and why their children use screen-based media*.

What technology and devices that involve screen time does your child have access to?

What do you know about Screen time related to children with autism spectrum disorder?

Can you tell me about how and why your child uses Screen time?

Which devices do they use mostly?

What do they use it for?

When do they use it?

How much of their day do they use it? Is it 30 min–2 h or 2–4 h or 4–8 h or more than 8 h per day?

Is screen time for your child different on the weekends and why?

What content do they access during screen time? Is it movies, you tube, games, learning apps, video conferencing, online schooling?

*Objective 2 &3: to identify and describe parents’ understandings/views of the effects of screen time on their children's lives.*

Can you tell me how you feel about your child's Screen time?

Can you tell me how does Screen time of your child benefit you and your child? Are there any benefits? What are the benefits to your family?

Can you tell me about how screen time negatively affects your child, and your family?

Do you think you need to manage the screen time of your child in any way? (If yes, how do you manage the screen time of your child?)

Can you tell me how Screen time affects your relationship and ability to engage with your child?

*Objective 4: To identify and describe how screen time has changed and the challenges around using screen-based media for their children during the COVID-19 pandemic.) to identify and describe changes in screen time, (including time spent, functionality and content) and challenges around using screen-based media for their children during the COVID-19 pandemic*.

Tell me about how screen time has changed for your child since the start of the COVID-19 pandemic.

What has changed and why?

Is your child using screen time more or less?

Has the content of what they use changed?

why are they using it?

What are the challenges now?

How has screen time been of benefit to your child during the pandemic?

Do you have any questions or comments for the researcher? would you like to know more about anything? if you think of anything after the interview that you would like to add, please make a note of it and let the researcher know at the next interview.

Schedule next interview date.

## References

[1] Slobodin O Heffler KF Davidovitch M. Screen media and autism spectrum disorder: a systematic literature review. J Dev Behav Pediatr. (2019) 40(4):303–11. 10.1097/DBP.0000000000000654 30908423

[2] Montes G. Children with autism spectrum disorder and screen time: results from a large, nationally representative US study. Acad Pediatr. (2016) 16(2):122–8. 10.1016/j.acap.2015.08.007 26525987

[3] Mazurek MO Television WC. Video game and social media use among children with ASD and typically developing siblings. J Autism Dev Disord. (2013) 43(6):1258–71. 10.1007/s10803-012-1659-9 23001767

[4] Jones RA Downing K Rinehart NJ Barnett LM May T McGillivray JA Physical activity, sedentary behavior and their correlates in children with autism spectrum disorder: a systematic review. PLoS One. (2017) 12(2):e0172482. 10.1371/journal.pone.0172482 28245224 PMC5330469

[5] Stiglic N Viner RM. Effects of screentime on the health and well-being of children and adolescents: a systematic review of reviews. BMJ Open. (2019) 9(1):e023191. 10.1136/bmjopen-2018-023191 30606703 PMC6326346

[6] Martins N King A Beights R. Audiovisual media content preferences of children with autism spectrum disorders: insights from parental interviews. J Autism Dev Disord. (2020) 50(9):3092–100. 10.1007/s10803-019-03987-1 30905049

[7] Larose V Sotelo K Mottron L Jacques C. Initial development of a questionnaire about parents’ perspectives on the strengths and interests of autistic preschoolers. Can J Behav Sci/Rev Can Sci Comport. (2021) 53(4):530–5. 10.1037/cbs0000268

[8] Stiglic N Viner RM. Effects of screentime on the health and well-being of children and adolescents: a systematic review of reviews. BMJ Open. (2019) 9(1):e023191. 10.1136/bmjopen-2018-023191 30606703 PMC6326346

[9] Mazurek MO Engelhardt CR Hilgard J Sohl K. Bedtime electronic media use and sleep in children with autism spectrum disorder. J Dev Behav Pediatr. (2016) 37(7):525–31. 10.1097/DBP.0000000000000314 27355885

[10] Ashburner J Rodger S Ziviani J Jones J. Occupational therapy services for people with autism spectrum disorders: current state of play, use of evidence and future learning priorities. Aust Occup Ther J. (2014) 61(2):110–20. 10.1111/1440-1630.12083 24118044

[11] Gwynette MF Sidhu SS Ceranoglu TA. Electronic screen media use in youth with autism spectrum disorder. Child Adolesc Psychiatr Clin N Am. (2018) 27(2):203–19. 10.1016/j.chc.2017.11.013 29502747

[12] Stiller A Mößle T. Media use among children and adolescents with autism spectrum disorder: a systematic review. Rev J Autism Dev Dis. (2018) 5(3):227–46. 10.1007/s40489-018-0135-7

[13] Stiller A Weber J Strube F Mößle T. Caregiver reports of screen time use of children with autism spectrum disorder: a qualitative study. Behav Sci (Basel, Switzerland). (2019) 9(5):56. 10.3390/bs9050056 PMC6562753 31121966

[14] Polfuss M Johnson N Bonis SA Hovis SL Apollon F Sawin KJ. Autism spectrum disorder and the child’s weight-related behaviors: a parents’ perspective. J Pediatr Nurs. (2016) 31(6):598–607. 10.1016/j.pedn.2016.05.006 27339734

[15] Nally B Houlton B Ralph S. Researches in brief: the management of television and video by parents of children with autism. Autism. (2000) 4(3):331–7. 10.1177/1362361300004003008

[16] Suresh A Tiwari S. Parental knowledge, attitudes and concerns towards media technology and screen time use in children with ASD and typically developing children. Technol Disabil. (2023) 35(1):21–42. 10.3233/TAD-220389

[17] Mello S Alper M Allen AA. Physician mediation theory and pediatric media guidance in the digital age: a survey of autism medical and clinical professionals. Health Commun. (2020) 35(8):955–65. 10.1080/10410236.2019.1598744 30947543

[18] Franz L Chambers N von Isenburg M de Vries P. Autism spectrum disorder in sub-saharan Africa: a comprehensive scoping review. Autism Res. (2017) 10(5):723–49. 10.1002/aur.1766 28266791 PMC5512111

[19] Guler J de Vries PJ Seris N Shabalala N Franz L. The importance of context in early autism intervention: a qualitative South African study. Autism. (2018) 22(8):1005–17. 10.1177/1362361317716604 28914083 PMC5832543

[20] Obrusnikova I Miccinello DL. Parent perceptions of factors influencing after-school physical activity of children with autism spectrum disorders. Adapt Phys Activ Q. (2012) 29(1):63–80. 10.1123/apaq.29.1.63 22190053

[21] Bradshaw C Atkinson S Doody O. Employing a qualitative description approach in health care research. Global Qual Nurs Res. (2017) 4:2333393617742282. 10.1177/2333393617742282 PMC5703087 29204457

[22] Franz L Adewumi K Chambers N Viljoen M Baumgartner JN de Vries PJ. Providing early detection and early intervention for autism spectrum disorder in South Africa: stakeholder perspectives from the western cape province. J Child Adolesc Ment Health. (2018) 30:149–65. 10.2989/17280583.2018.1525386 30403918 PMC6301128

[23] Pillay S Duncan M de Vries P. How many children with autism spectrum disorder are there in South African schools: a systematic database search for known cases of ASD in the western cape. Poster Presented at: Regional IMFAR SA-ACAPAP2017 (2017).

[24] Yin RK. Qualitative Research from Start to Finish. 2nd edn. New York: Guilford Publications (2015).

[25] Descript. Descript for transcription (2021). Available online at: https://www.descript.com/ (Accessed May 11, 2025).

[26] Vaismoradi M Snelgrove S. Theme in qualitative content analysis and thematic analysis. Forum Qual Soz/Forum Qual Soc Res. (2019) 20(3):23. https://nordopen.nord.no/nord-xmlui/bitstream/handle/11250/2627867/Vaismoradi.pdf

[27] General Assembly of the World Medical Association. World medical association declaration of Helsinki: ethical principles for medical research involving human subjects. J Am Coll Dent. (2014) 81(3):14. https://pubmed.ncbi.nlm.nih.gov/25951678/25951678

[28] Ebrahim FO. Parent perceptions of screen time use in young children with autism Spectrum disorder (Master’s thesis). University of Cape Town (2022). Available online at: https://open.uct.ac.za/server/api/core/bitstreams/58258190-7b16-4668-a490-6faeb8eac582/content

[29] Valkenburg PM Krcmar M Peeters AL Marseille NM. Developing a scale to assess three styles of television mediation: “instructive mediation,” “restrictive mediation,” and “social coviewing”. J Broadcast Electron Media. (1999) 43(1):52–66. 10.1080/08838159909364474

[30] Warren R. In words and deeds: parental involvement and mediation of children’s television viewing. J Fam Commun. (2001) 1(4):211–31. 10.1207/S15327698JFC0104_01

[31] Kuo MH Magill-Evans J Zwaigenbaum L. Parental mediation of television viewing and videogaming of adolescents with autism spectrum disorder and their siblings. Autism. (2015) 19(6):724–35. 10.1177/1362361314552199 25336095

[32] Dong H-Y Feng J-Y Wang B Shan L Jia F-Y. Screen time and autism: current situation and risk factors for screen time among pre-school children with ASD. Front Psychiatry. (2021) 12:675902. 10.3389/fpsyt.2021.675902 34421670 PMC8377252

[33] Nikken P Schols M. How and why parents guide the media use of young children. J Child Fam Stud. (2015) 24(11):3423–35. 10.1007/s10826-015-0144-4 26472932 PMC4598347

[34] Rivard M Terroux A Parent-Boursier C Mercier C. Determinants of stress in parents of children with autism spectrum disorders. J Autism Dev Disord. (2014) 44(7):1609–20. 10.1007/s10803-013-2028-z 24384673

[35] Colizzi M Sironi E Antonini F Ciceri ML Bovo C Zoccante L. Psychosocial and behavioral impact of COVID-19 in autism spectrum disorder: an online parent survey. Brain Sciences (2076–3425). (2020) 10(6):341. 10.3390/brainsci10060341 32503172 PMC7349059

[36] Jacques C Saulnier G Éthier A Soulières I. Experience of autistic children and their families during the pandemic: from distress to coping strategies. J Autism Dev Disord. (2021) 52(8):3626–38. 10.1007/s10803-021-05233-z 34448994 PMC8391854

[37] Boeren E. Understanding sustainable development goal (SDG) 4 on “quality education” from micro, meso and macro perspectives. Int Rev Educ. (2019) 65(2):277–94. 10.1007/s11159-019-09772-7

[38] Bozoglan B Kumar S. Parenting styles, parenting stress and hours spent online as predictors of child internet addiction among children with autism. J Autism Dev Disord. (2021) 52(10):4375–83. 10.1007/s10803-021-05324-x 34647155

[39] Aarthun A Øymar K Akerjordet K. Parental involvement in decision-making about their child’s health care at the hospital. Nurs Open. (2018) 6:50–8. 10.1002/nop2.180 30534394 PMC6279730

